# Simvastatin accelerated motoneurons death in SOD1^G93A^ mice through inhibiting Rab7-mediated maturation of late autophagic vacuoles

**DOI:** 10.1038/s41419-021-03669-w

**Published:** 2021-04-12

**Authors:** Lin Bai, Yafei Wang, Jia Huo, Shuai Li, Ya Wen, Qi Liu, Jing Yang, Yaling Liu, Rui Li

**Affiliations:** 1 Department of Neurology, The Second Hospital of Hebei Medical University, Shijiazhuang, Hebei, 050000 P.R. China; 2 Neurological Laboratory of Hebei Province, Shijiazhuang, Hebei, 050000 P.R. China

**Keywords:** Amyotrophic lateral sclerosis, Amyotrophic lateral sclerosis

## Abstract

Amyotrophic lateral sclerosis (ALS) is a progressive neurodegenerative disease caused by motoneuron loss, for which there is currently no effective treatment. Statins, as inhibitors of 3-hydroxy-3-methylglutaryl-CoA (HMG-CoA) reductase, are used as drugs for treatment for a variety of disease such as ischemic diseases, neurodegenerative diseases, cancer, and inflammation. However, our previous evidence has demonstrated that simvastatin leads to cytotoxicity in NSC34-hSOD1^G93A^ cells by aggravating the impairment of autophagic flux, but the role of simvastatin in ALS model remains elusive. In present study, we reported that after simvastatin treatment, SOD1^G93A^ mice showed early onset of the disease phenotype and shortened life span, with aggravated autophagic flux impairment and increased aggregation of SOD1 protein in spinal cord motoneurons (MNs) of SOD1^G93A^ mice. In addition, simvastatin repressed the ability of Rab7 localization on the membrane by inhibiting isoprenoid synthesis, leading to impaired late stage of autophagic flux rather than initiation. This study suggested that simvastatin significantly worsened impairment of late autophagic flux, resulting in massive MNs death in spinal cord and accelerated disease progression of SOD1^G93A^ mice. Together, these findings might imply a potential risk of clinic application of statins in ALS.

## Introduction

Amyotrophic lateral sclerosis (ALS) is a rare progressive neurodegenerative disease characterized by the selective demise of motoneurons (MNs). Muscle weakness and atrophy are symptoms that ultimately lead to respiratory failure and death, usually within 3–5 years^1^. Approximately 90% of ALS cases are sporadic (sALS), whereas the remaining 10% are familial (fALS). Moreover, ~20% of fALS and 5% of sALS cases are attributed to mutations in the Cu/Zn-superoxide dismutase (*SOD1*) gene^2,3^. Although several possible pathogenic mechanisms, including protein aggregation, mitochondrial dysfunction, impaired RNA processing and metabolism, neurotrophic factor deficiency, and axoplasmic transport abnormality^4–6^ are currently reported, the potential causes of ALS remains poorly understood.

Macroautophagy (hereafter autophagy) is an important lysosomal degradation route in which cytosolic components, especially long-lived or misfolded/aggregate-prone proteins, damaged whole organelles, are effectively removed and recycled^7^. Autophagy involves the sequestration of cytoplasmic materials into a double-membrane autophagosomes (APs) and subsequent delivery to lysosomes for catabolism^8^. In fact, nascent APs are capable of trafficking to the lysosome and fusing with early or late phase endosomes/lysosomes and subsequent forming degradative autolysosomes, a process known as AP maturation^8^. The small GTP binding protein Rab7 is a member of the Rab family that has been required for late endosomal/autophagosomal transport and finally participated in the fusion step with lysosomes^9–11^. Neurons appear to be particularly dependent on autophagy^12^. Aberrant autophagy including hyperactive autophagy induction, defective AP formation, or dysfunction of AP maturation could result in protein aggregation and finally neuronal death. Abnormal autophagy has been found in several neurodegenerative diseases such as Alzheimer’s disease (AD) and Parkinson’s disease (PD), as well as ALS^3,13–15^.

The mevalonate (MVA) pathway is essential for sterols synthesis, especially cholesterol and also plays a key role in producing isoprenoids, such as farnesyl pyrophosphate (FPP) and geranylgeranyl pyrophosphate (GGPP), which are necessary sources for protein prenylation. In particular, prenylation comprises an important posttranslational process that modifies a variety of proteins, such as the family of small GTPase proteins (Rabs and Ras family), and ensures their correct localization on membranes^16^. MVA pathway affects various metabolism pathways, such as protein synthesis and degradation, intracellular signaling, cell growth, and differentiation or death^17–19^. Furthermore, several studies reported that severe reduction of protein prenylation by MVA pathway blockade has been linked to defective autophagy^17,20^.

Statins act as inhibitors of 3-hydroxy-3-methylglutaryl-CoA (HMG-CoA) reductase (HMGCR), a key rate-limiting enzyme in the MVA pathway, which catalytically converts HMG-CoA into MVA. By lowing LDL levels, statins have been widely used in the clinic for treating hypercholesterolemia, cardiovascular, and cerebrovascular disease^21–23^. Moreover, the pleiotropic effects (independent cholesterol) of statins have a protective effect in many diseases, including AD, PD, and multiple sclerosis (MS)^24–26^. However, accumulating data have suggested that statins increased risk of diabetes, liver disorders, or myopathy^27–29^. Thus, the role of statins remains controversial. Some patients with ALS exhibited the symptoms of cardiovascular and cerebrovascular disease, and it remains unclear whether statin treatment is detrimental for ALS.

We have shown previously that simvastatin increased cytotoxic effects on NSC34-hSOD1^G93A^ cells, which could be attributed to the aggravated impairment of autophagic flux by reducing synthesis of isoprenoids rather than cholesterol^30^. Therefore, to further clarify the detailed effect of statin in vivo, we used ALS mice model through specific mutation of *SOD1*. Here, we showed that statins inhibited activity of Rab7 via reduction of FPP and GGPP in the MVA pathway, which in turn further aggravated defect of late autophagic flux and finally led to massive neuronal death in SOD1^G93A^ mice.

## Materials and methods

### Chemicals

Simvastatin (Cat. No: S6196), FPP (Cat. No: F6892), GGPP (Cat. No: G6025), and CID1067700 (SML0545) were purchased from Sigma-Aldrich (St. Louis, MO, USA). Sodium carboxymethyl cellulose (CMC-Na, Cat. No: C8621). FPP and GGPP are liquid format. Simvastatin was dissolved in CMC-Na. CID1067700 was dissolved in dimethyl sulfoxide (DMSO).

### Animals and treatments

Transgenic SOD1^G93A^ mice (B6SJL-Tg [SOD1G93A] 1Gur/J) were originally obtained from Jackson Laboratory (Bar Harbor, ME, USA). The mice were maintained as hemizygotes by crossing transgenic males with wild type (WT) B6SJL/F1 females. Hemizygous mice were genotyped by PCR according to our previous protocol^31^. The mice were housed in controlled temperature and humidity facility (light/dark cycle 12/12 h), while were fed sterile water and sterilized specific pathogen-free rodent food at will. Our preliminary study demonstrated that female SOD1^G93A^ mice showed more significant behavioral changes after administration of 20 mg/kg simvastatin, thus determining the gender of mice and the dosage of simvastatin (20 mg/kg). Simvastatin dissolved in 5% CMC-Na vehicle, then given by oral gavage once a day for mice. The SOD1^G93A^ mice have no symptoms or obvious pathological changes in spinal cord at 60 days. Thus, simvastatin treatment was started from 63 days (9th week) to the endstage. No obvious side effects were observed during the course of treatment. Female SOD1^G93A^ mice were randomized into two groups: Simvastatin group (G93A-Sim); Vehicle group (G93A-Con). Simultaneously, female-matched WT littermates were divided into WT-Sim and WT-Con groups. Each group of 18 mice was used to assess disease onset and life span. Additionally, the other SOD1^G93A^ mice and WT littermates were also treated from 63 days and sacrificed at 120 days of age. After treatments, the rest of the mice were subjected to histological analysis or Western blot analysis (five mice per group) as described below. Other mice were used for time point analysis (90 days, endstage; five mice per group). All animal experiments were conducted according to the management regulations of laboratory animal promulgated by the Second Hospital of Hebei Medical University. All experiments were approved by the Research Ethics Committee of the Second Hospital of Hebei Medical University (Shijiazhuang, Hebei, P.R. China, Approval No. 2020P023).

### Assessment of disease onset and life span

In order to assess the motor functions of mice, we performed three clinical tests: (i) weighed, (ii) performed the rotarod test, and (iii) scored of neurological deficits. All of these tests are usually used to evaluate SOD1^G93A^ mice^32^.

Body weight was measured once per week, starting at 63 days (9th week) of age. Assessed the disease onset of SOD1^G93A^ mice by rotarod test. After 5-day training session, the rotarod test was performed at a constant speed (Rotarod test apparatus: 4 cm diameter, 20 rpm). The tests started from ^9th^ week for once a week, then from ^12th^ week for twice a week. All mice were measured three times (5 min/trial), 10-min interval for each test. When the mice could not insist 5 min for the longest run time at all three trials that will be recorded to the disease onset. Scored of neurological deficits as following four-point scoring system^33^. Score of 4: normal (no sign of motor dysfunction); Score of 3: hind limb tremors are evident when suspended by the tail; Score of 2: gait abnormalities are present; Score of 1: drag of at least one hind limb; Score of 0: inability to right itself within 30 s. For the life span, the inability of SOD1^G93A^ mice to right themselves within 30 s of being place on their back was defined as the date of “end stage”.

### Histology

Mice were deeply anesthetized by chloral hydrate and transcardially perfused by phosphate buffered saline (PBS, PH7.4) and followed by ice-cold 4% paraformaldehyde (PFA) in PBS. Spinal cords were harvested then postfixed in 4% PFA overnight, and then equilibrated in 10 and 30% sucrose/PBS at 4 °C for 24 h, respectively. Lumbar spinal cords were embedded in O.C.T. medium, then consecutive cut in 8 μm transverse sections by cryostat microtome. For MNs counting, sections were stained with 1% cresyl violet using a standard protocol, dehydrated in gradient alcohol, and cleared in xylol. Nissl-positive MNs were counted from ventral horns per mouse (five mice per group), which considered as neuronal morphology, diameter exceeding 8 μm and a distinct nucleolar profile. We observed the sample slides, photographed by microscope (Olympus, BX51, Tokyo, Japan) and counted MNs in the ventral horns of both sides. The MNs were counted by independent investigator who was blinded to groups.

### Immunofluorescence Staining

The lumbar spinal cords (L4-L5) at 120 days of age were obtained for immunofluorescent staining. Lumbar spinal cords were dissected and cut into 20 μm transverse sections. The frozen sections were placed for 20 min at room temperature, and then washed with PBS for three times (5 min/time). Tissue sections were blocked in 10% goat or donkey serum in PBS-0.3% Triton X-100 for 1 h at room temperature and incubated with primary antibodies against CHAT antibody (1:1000, Abcam, ab178850), NeuN (1:500, CST, #24307 s), GFAP (1:500, CST, #3670 s), Iba1 (1:500, Abcam, ab178847), LC3 (1:200, Santa, sc-376404), P62 (1:500, Sigma, P0067), LAMP2 (1:200, Abcam, ab25631), LC3 (1:500, Sigma, L7543), SOD1 (1:200, Abcam, ab16831), or Rab7 (1:200, Abcam, ab137029) overnight at 4 °C. Then sections were washed in PBS for three times and incubated with Alexa Fluor 488-conjugated Goat anti-Rabbit secondary antibody (1:1000, Thermo Fisher, #A-11034), Alexa Fluor 594-conjugated Goat anti-Mouse secondary antibody (1:1000, Thermo Fisher, #A-11032), or Alexa Fluor 647-conjugated Donkey anti-Goat secondary antibody (1:1000, Thermo Fisher, #A211447) for 1 h at room temperature. After washing (PBS, three times), nuclei were counter stained with DAPI Fluoromount-G (Southern Biotech). The sections were visualized with a fluorescence confocal microscope (Olympus FV1000). The parameters of experimental setup are determined at the beginning of each individual imaging process and remain constant throughout the imaging process. The mean number, including LC3 or Rab7 puncta/cell, colocation of P62/LC3 or LC3/LAMP2, NeuN/Rab7, GFAP/Rab7, Iba1/Rab7 and CHAT was counted by an investigator blinded to groups. The mean fluorescence density of P62, SOD1, GFAP, or Iba1 was analyzed with ImageJ. The above experiments were repeated three times with five mice in each group.

### Western blotting

Proteins were extracted using a bicinchoninic acid (BCA) protein assay kit (Thermo Fisher Scientific, 23225, Waltham, MA, USA). Equal amount of proteins (40 μg) were separated by 10 or 12% SDS-PAGE and then transferred to PVDF membranes. After blocking by 5% nonfat milk, the membranes were incubated with primary antibodies overnight at 4 °C, and then washed three times (10 min/time), subsequently with secondary antibodies for 1 h at room temperature. Finally, the bands on the membranes were scanned with an Odyssey Infrared Imaging System (LI-COR, Lincoln, NE, USA), and quantities analysis was performed with ImageJ.

The primary antibodies: P62 (1:1000, Sigma, P0067), LC3 (1:1000, Sigma, L7543), SOD1 (1:2000, Abcam, ab16831), HMGCR (1:200, Santa, sc-271595), FDPS (1:100, Proteintech, 16129-1), RABGGTA (1:100, Proteintech, 14448-1), Rab7 (1:500, Abcam, ab137029), and β-actin (1:1000, Proteintech, 60008-1).

### Electron microscopy

Mice were deeply anesthetized and follow perfused with 4% PFA and 2.5% glutaraldehyde in PBS. The spinal cord was dissected and postfixed in 4% glutaraldehyde at 4 °C for 24 h. After the tissues were washed three times, they were postfixed with 1% osmium tetroxide for 2 h, and then stained with 1% uranyl acetate for 1 h. Next, the tissues were embedded in epoxy resin after being dehydrated by gradient ethanol. Ultra-thin sections (70 nm thick) were prepared for electron microscopy analysis. The ultra-thin sections were observed and imaged under a transmission electron microscopy (TEM, JEM-1230). Five mice were used at each group, taken the lumbar spinal cords for thin sections from each mouse. Five sections were cut per tissue. Ten electron micrographs taken from the neurons of the anterior crus of lumbar spinal cord were taken per section. Morphometrical measurements of APs were carried out using the point-counting method^34–36^. The number of APs in the cytoplasm of MNs in WT and SOD1G93A mice groups were counted by an investigator blinded to groups and compared.

### NSC34 cell culture and transfection

Mouse neuroblastoma x spinal cord (NSC34) cells retain the ability of proliferation and exhibit the characteristics of MNs^37^. NSC34 MN-like cells stably expressing GFP-empty vector (E), GFP-human SOD1 wild type (hSOD1^WT^), or GFP-human SOD1G93A (hSOD1^G93A^) constructs were generated as previously described in our laboratory^38^. For all experiments, the cells were starved in serum-free Dulbecco’s modified Eagle’s medium (DMEM) for 24 h, and then incubated in medium containing simvastatin (1 µM) alone or coculture FPP (10 µM) or GGPP (10 µM) for 24 h. For the CID1067700 (CID) experiments, NSC34-E and NSC34-hSOD1^G93A^ cells were treated with CID (40 μM) for 2 h, or NSC34-hSOD1^G93A^ cells were incubated with GGPP (10 μM) for 24 h and CID (40 μM) was added to the cells in the last 2 h before harvesting.

### Cell lysis and immunoblotting

Cells were lysed in IP buffer containing phenylmethylsulfonylfluoride (PMSF), and proteinase inhibitors for 30 min on ice. The cell lysates were centrifuged at 10,000 rpm for 10 min at 4 °C to collect supernatants. Protein concentrations were measured with BCA protein assay kit. Proteins were electrophoresed and transferred to PVDF membranes and incubated with antibody. Finally, the bands were quantified using ImageJ software. The following primary antibodies: P62 (1:1000, Sigma, P0067), LC3 (1:1000, Sigma, L7543), SOD1 (1:2000, Abcam, ab16831), HMGCR (1:200, Santa, sc-271595), FDPS (1:100, Proteintech, 16129-1), RABGGTA (1:100, Proteintech, 14448-1), Rab7 (1:500, Abcam, ab137029), and β-actin (1:1000, Proteintech, 60008-1).

#### Extraction of membrane protein and cytosol protein

Cells were treated as above described and collected on ice, and then isolated membrane protein and cytosol protein of cells according to the introduction of Minute^TM^ plasma membrane protein isolation kit (Invent Biotechnologies, SM-005, USA) instructions.

### Cell staining and imaging

Cells were grown on coverslips in 12-well plates (1 × 200 cells/well) overnight and then treated with corresponding chemicals that was performed as previously described. After that, cells were postfixed with 4% PFA for 20 min, then washed with PBS for three times (10 min/time), and then treated with 10% goat or donkey serum diluted in PBS-0.3% Triton X-100 for 30 min at room temperature. Next, cells were incubated with antibodies with P62 (1:500, Sigma, P0067), LC3 (1:200, Santa, sc-376404), LAMP2 (1:200, Abcam, ab25631), LC3 (1:500, Sigma, L7543), SOD1 (1:400, Abcam, ab16831), or Rab7 (1:500, Abcam, ab137029) overnight at 4 °C. The cells were washed with PBS for three times and stained with secondary antibodies, Alexa Fluor 594-conjugated goat anti-rabbit (1:1000, Thermo Fisher, #A-11037) or Alexa Fluor 647-conjugated goat anti-mouse (1:1000, Thermo Fisher; #A-21236), for 1 h at room temperature, then stained nuclei with DAPI Fluoromount-G (Southern Biotech). Finally, imaged the cells by confocal microscopy.

#### Filipin staining

Cells were processed as described. After 4% PFA postfixed and PBS rinsed, cells were incubated with 1 ml glycine (1.5 mg/ml PBS) for 10 min. Then, cells were stained with 0.3 mg/ml Filipin (Sigma, SAE0088) in PBS (working solution) for 2 h at room temperature. Intracellular free cholesterol was stained by Filipin and fluoresced blue (excitation of 340–380 nm and emission of 385–470 nm).

### Statistical methods and analyses

Data were obtained from three independent experiments. The comparisons of disease onset and life span among groups were statistically analyzed by Kaplan–Meier survival analysis. Unpaired *t*-test was used to examine significant differences between two groups. Other statistical significances were evaluated by one-way ANOVA test to obtain multiplicity adjusted *p* values. Significance being defined as *P ≤ 0.05, **P ≤ 0.01, ***P ≤ 0.001; ^#^*P* ≤ 0.05, ^##^*P* ≤ 0.01, ^###^*P* ≤ 0.001. Statistical analysis was performed by GraphPad software. Data were expressed as the mean ± SEM.

## Results

### Simvastatin aggravated impairment of autophagic flux in SOD1^G93A^ mice

**Fig. 1:**
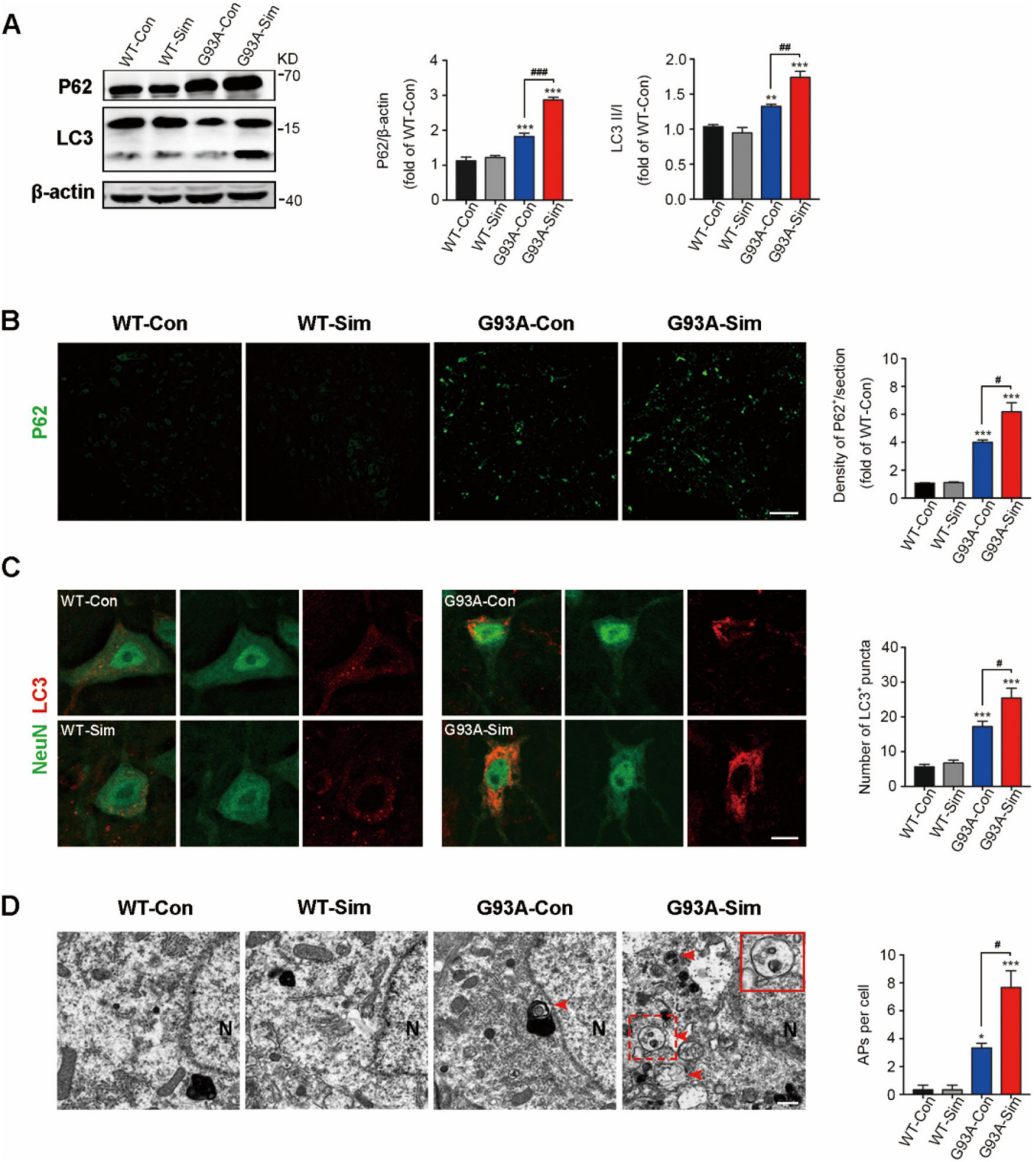
Simvastatin aggravated impairment of autophagic flux in SOD1^G93A^ mice. **A** Western blot analysis of P62 and LC3 in the lumbar spinal cords of WT and SOD1^G93A^ mice treated with or without simvastatin at 120 days. Quantification of P62 and LC3 levels from immunoblots normalized to the WT-Con group. **B** Immunofluorescence labeling of P62 (green) in the lumbar spinal cord at 120 days (Scale bar, 100 μm). Quantification of the average density of P62-positive cells per section. **C** Immunofluorescence labeling of LC3 (red) in NeuN-positive motoneurons (green) at 120 days (Scale bars, 10 μm). Quantitative analysis of LC3 puncta. **D** TEM micrographs showed that APs are present in the cytoplasm of MNs in SOD1^G93A^ mice at 120 days and that these structures are absent or rare in MNs of WT mice (red arrowheads; N, nucleus; scale bar, 1 µm). The upper right image shows magnifications for the red dotted box, as shown in the red solid line box. The histogram shows the quantification of APs per cell. (Data represent the mean ± SEM, *n* = 5 mice per group; statistical significance was assessed by one-way ANOVA or an unpaired *t*-test, *P ≤ 0.05, **P ≤ 0.01, ***P ≤ 0.001, ^#^*P* ≤ 0.05, ^##^*P* ≤ 0.01, ^###^*P* ≤ 0.001).

We firstly detected the levels of P62 and LC3, the classic markers to evaluate ability of autophagic flux, in the lumbar spinal cords of SOD1^G93A^ mice at 120 days. We found that P62 and LC3 are expressed at higher level in SOD1^G93A^ mice than those in WT mice, which was consistent with previous studies^15^. Importantly, in mice treated with simvastatin (G93A-Sim group), both of P62 and LC3 expression were increased significantly compared with control (G93A-Con) group, but there is no significant difference between WT-Con and WT-Sim groups (Fig. 1A). In addition, lots of P62 immunoreactive puncta were detectable in the lumbar spinal cords of SOD1^G93A^ mice, and these aggregates were extremely increased by long-term simvastatin treatment (Fig. 1B). Notably, expression of LC3 was enhanced in the NeuN-positive MNs of G93A-Con compared with WT, and G93A-Sim group displayed higher LC3 aggregation. However, no significant difference was observed between WT-Con and WT-Sim groups (Fig. 1C). Moreover, a large number of closed double-membrane-bound APs were observed by TEM analysis in the cytoplasm of MNs in G93A-Con mice, and the number of Aps were remarkably upregulated after treated with simvastatin (Fig. 1D, as shown by arrowheads). These results suggested that simvastatin was able to further aggravate the disruption of autophagic flux in SOD1^G93A^ mice, and this effect might be linked to early enhanced autophagy induction or defective degradation at the late stage.

### Simvastatin increased the aggregation of the SOD1 protein in the lumbar spinal cords of SOD1^G93A^ mice

**Fig. 2:**
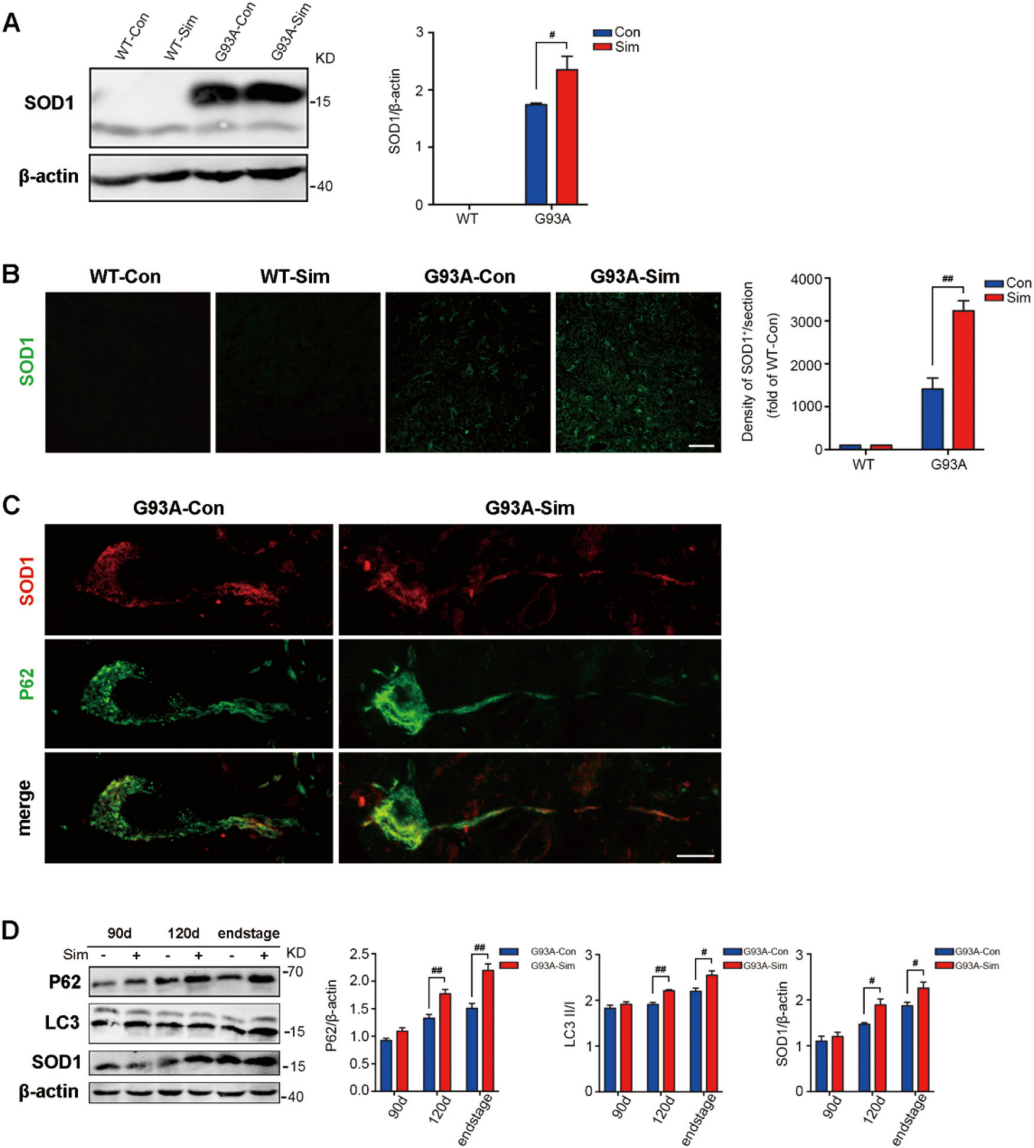
Simvastatin increased SOD1 aggregation in SOD1^G93A^ mice. **A** Western blot analysis of SOD1 levels in the lumbar spinal cord at 120 days. Quantitative analysis of SOD1 levels. **B** Immunofluorescence labeling of SOD1 (green) in the lumbar spinal cord at 120 days (Scale bars, 50 μm). Quantification of SOD1 immunoreactivity. **C** Colocalization of SOD1 (red) and P62 (green) in MNs of G93A-Sim and G93A-Con mice at 120 days (Scale bars, 10 μm). **D** Representative Western blot quantification of P62, LC3, and SOD1 levels in the lumbar spinal cord of G93A-Sim and G93A-Con mice at 90 days, 120 days, and end-stage of disease. (Data represent the mean ± SEM, *n* = 5 mice per group; statistical significance was assessed by one-way ANOVA or an unpaired *t*-test, ^#^*P* ≤ 0.05, ^##^*P* ≤ 0.01, ^###^*P* ≤ 0.001).

Misfolding and aggregation of mutant SOD1 (mSOD1) is a pathological hallmark of a subset of fALS patients and is involved in pathogenesis^3,39,40^. As expected, compared with WT group, increased expression of SOD1 protein was detectable in the lumbar spinal cords of G93A-Con mice at 120 days, and was drastically elevated after simvastatin administration (Fig. 2A). Consistent with the western blot data, amount of SOD1 aggregation were significantly upregulated in SOD1^G93A^ mice treated with simvastatin by immunostaining (Fig. 2B). We further found the higher abundance of autophagic vacuoles labeled with SOD1 colocalized with P62, the ubiquitin-binding domain-containing receptor in G93A-Sim mice compared with G93A-Con (Fig. 2C). Next, we compared protein levels of P62, LC3, and SOD1 in the lumbar spinal cords of SOD1^G93A^ mice treated or untreated with simvastatin over the course of disease progression. All of the expression of the P62, LC3, and SOD1 proteins in the G93A-Sim group showed dramatical increase from 120 days to the end stage compared to G93A-Con (Fig. 2D). These results indicated that simvastatin extensively enhanced the level of SOD1 aggregation in G93A-Con mice associated with increased defect of autophagic flux.

### Simvastatin accelerated neuron death and disease phenotype onset in SOD1^G93A^ mice

**Fig. 3:**
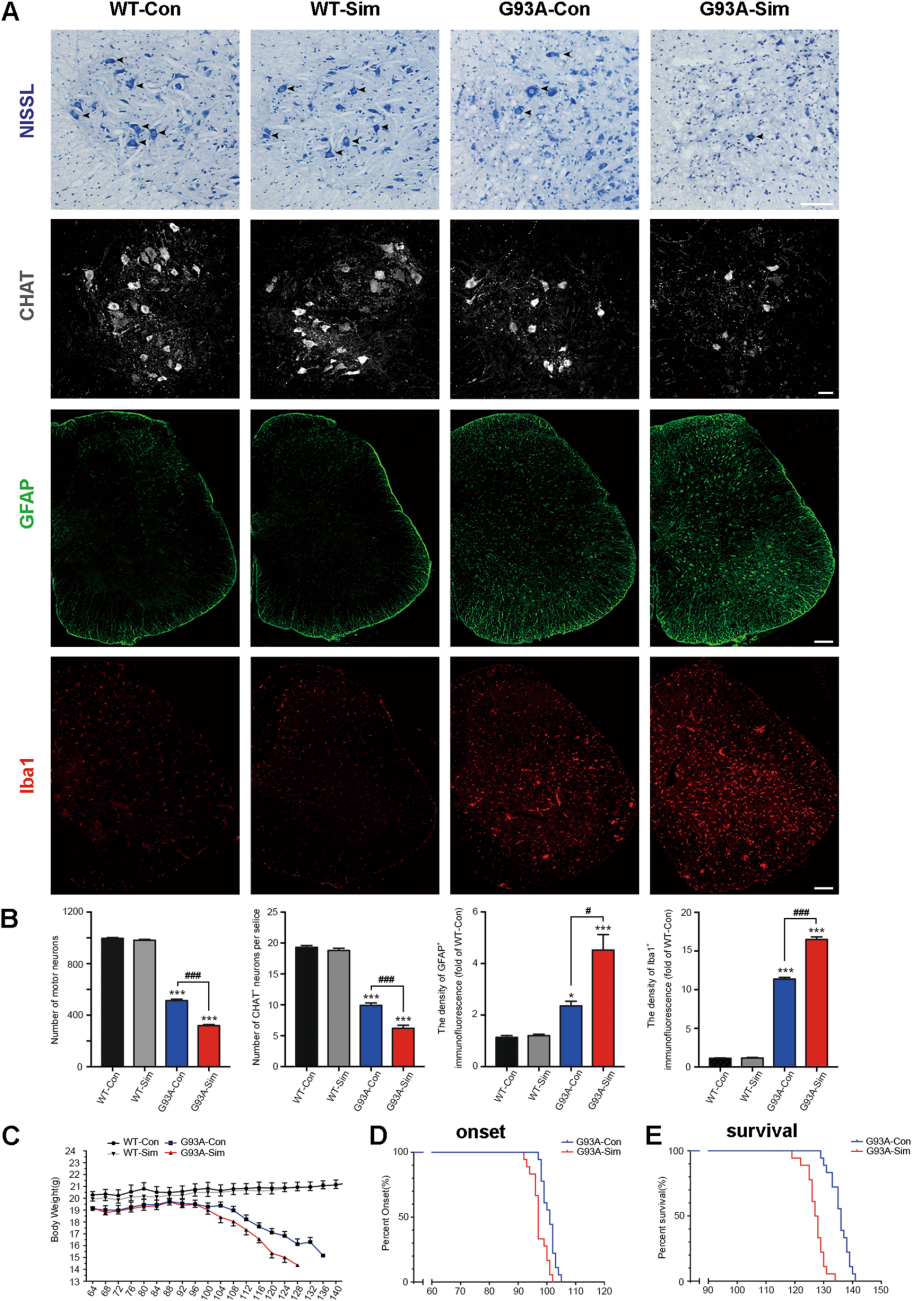
Simvastatin accelerated neuron death and onset of the disease phenotype in SOD1^G93A^ mice. **A** Photographs of Nissl-stained MNs in the ventral horn of the lumbar spinal cord at 120 days. Immunofluorescence labeling of CHAT, GFAP, and Iba1 in the lumbar spinal cord at 120 days. (Scale bars, 100 μm). **B** The number of MNs in the lumbar spinal cord in different groups. Quantification of CHAT positive cells in the lumbar spinal cord. Quantification of GFAP intensity in the lumbar spinal cord. Quantification of Iba1 intensity in the lumbar spinal cord. (Data represent the mean ± SEM, *n* = 5 mice per group; statistical significance was assessed by one-way ANOVA or an unpaired *t*-test, *P ≤ 0.05, **P ≤ 0.01, ***P ≤ 0.001, ^#^*P* ≤ 0.05, ^##^*P* ≤ 0.01, ^###^*P* ≤ 0.001). **C**–**E** Kaplan–Meier plots showing the effects of simvastatin treatment on disease phenotypes. **C** The body weight curves of WT and SOD1^G93A^ mice treated or untreated with simvastatin. Simvastatin accelerates body weight loss in SOD1^G93A^ mice. **D** The probability of disease onset for G93A-Sim relative to G93A-Con. **E** The probability of survival for G93A-Sim relative to G93A-Con. Compared to G93A-Con mice, G93A-Sim mice display earlier disease onset and shortened survival. (Data represent the mean ± SEM, *n* = 18 mice per group; *P ≤ 0.05, **P ≤ 0.01, ***P ≤ 0.001, ^#^*P* ≤ 0.05, ^##^*P* ≤ 0.01, ^###^*P* ≤ 0.001).

Given the appearance of disease phenotype is closely related to mSOD1 accumulation in the spinal cord and brainstem of mice^41^. It has been posited that aggregation and/or misfolding of SOD1 is one of the most likely causes of neurotoxicity, revealing a potential mechanism for MN impairment^42–44^. We quantified the number of MNs in lumbar spinal cord sections from each group by Nissl staining. The abundance of MNs reduced 46.7% in the lumbar spinal cords of SOD1^G93A^ mice compared to WT mice at 120 days (*p* < 0.001; Fig. 3A, arrowheads, 3B). In particular, simvastatin treatment exacerbated MN loss by ~20.1% relative to G93A-Con group (*p* < 0.001; Fig. 3A, B). Immunofluorescence staining of CHAT also indicated an adverse impact of simvastatin on MN loss in the lumbar spinal cords of SOD1^G93A^ mice (*p* < 0.001; Fig. 3A, B). These results suggested that simvastatin induced massive MN loss in the lumbar spinal cords accompanied by severe aggregation of SOD1. As a comparison with MNs, we next investigate activation of the inflammatory glial cells, such as reactive astrocyte and microglia. Simvastatin caused a dramatical increase in the quantity of both GFAP-positive astrocytes and Iba1-positive microglia cells in the whole lumbar spinal cords of SOD1^G93A^ mice at 120 days. Meanwhile, immunostaining for GFAP and Iba1 revealed a significant upregulation in SOD1^G93A^ mice compared to WT. But there was no significant change in WT-Con relative to WT-Sim (Fig. 3A, B).

As body weight loss is a frequent clinical symptom of patients with ALS^1^. We then monitored G93A-Sim mice and found the body weight of these mice was severely decreased compared to G93A-Con during disease progression, whereas body weight was slightly increased in both WT-Con and WT-Sim groups (Fig. 3C). Moreover, compared with G93A-Con mice, G93A-Sim mice showed early onset of disease, as assessed by the rotarod test (97.33 ± 2.77 d vs 100.72 ± 2.32 d, *P* < 0.001; Fig. 3D). Simvastatin treatment also significantly shortened the life span of SOD1^G93A^ mice (127.22 ± 3.37 d vs 135.61 ± 3.40 d, *P* < 0.001; Fig. 3E). Taken together, these results demonstrated that simvastatin accelerated neuronal death and earlier onset of the disease phenotype, finally severely shorten survival of SOD1^G93A^ mice.

### Simvastatin blocked the late stage of autophagic flux rather than early initiation

**Fig. 4:**
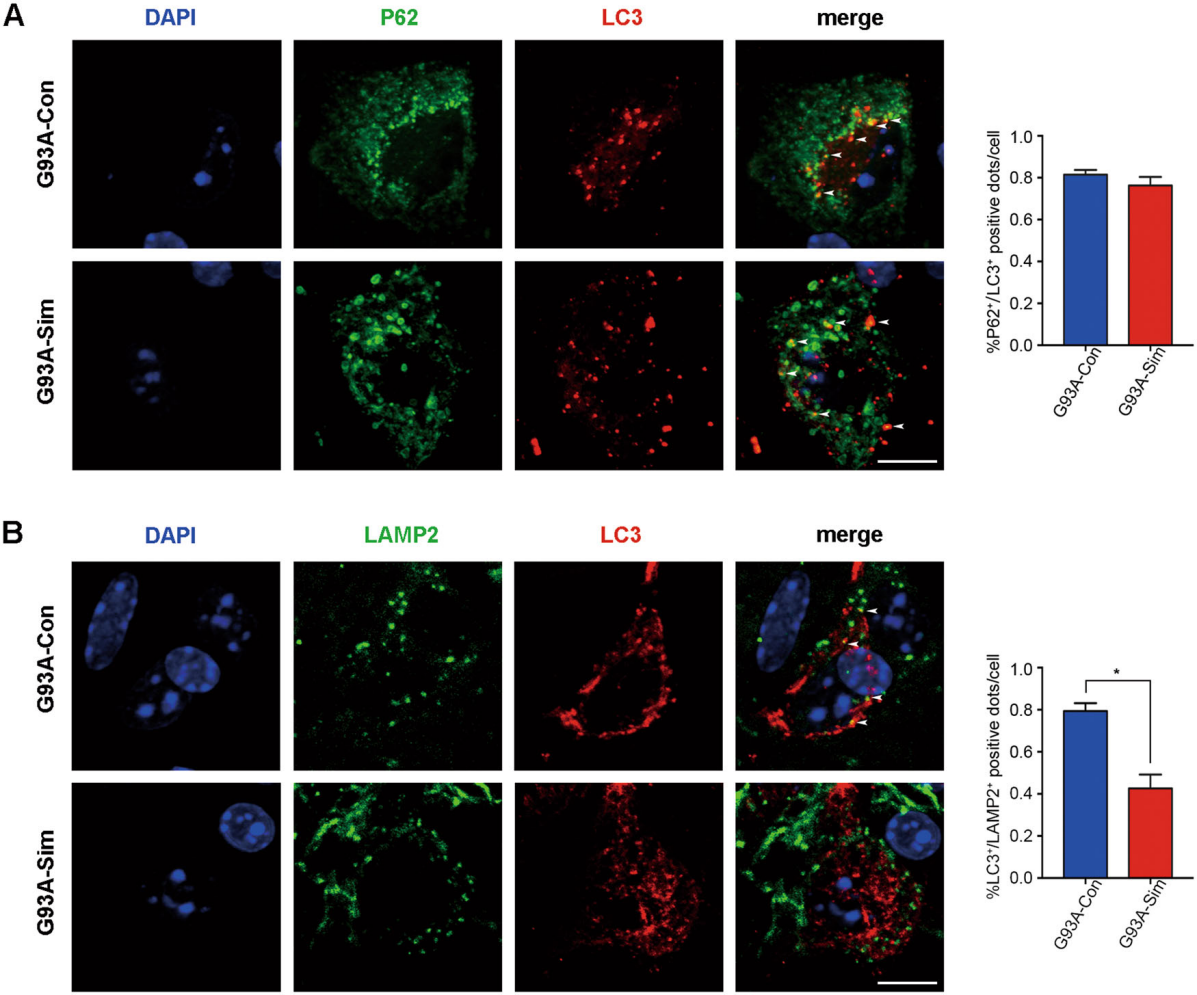
Simvastatin blocked the maturation of autophagosomes in spinal MNs of SOD1^G93A^ mice. **A** Immunofluorescence labeling of P62 (green), LC3 (red), and DAPI (blue) in the lumbar spinal cord MNs of G93A-Sim and G93A-Con mice at 120 days, respectively (Scale bars, 10 μm). White arrowheads show the colocalization of P62 and LC3. Analysis of the ratio of colocalization of P62 and LC3. **B** Immunofluorescence labeling of LAMP2 (green), LC3 (red), and DAPI (blue) in MNs at 120 days (Scale bars, 10 μm). White arrowheads show the colocalization of LAMP2 and LC3. Analysis of the ratio of colocalization of LAMP2 and LC3. (Data represent the mean ± SEM, *n* = 5 mice per group; statistical significance was assessed by an unpaired *t*-test, *P ≤ 0.05).

To determine at what stage dose the blockage of autophagic flux occur, we firstly double labeled p62-LC3. Autophagy substrates (p62/SQSTM1) directly binds to the AP membrane protein LC3 and is selectively degraded by autophagy, whereas LC3 and associating p62/SQSTM1 which localized on both outer and inner membranes^3,45–47^. To evaluate the fusion of P62 with LC3, a marker of AP formation, we quantified the percent of P62- and LC3-double positive structures and found no significant difference between the lumbar spinal MNs of G93A-Con and G93A-Sim at 120 days (Fig. 4A, arrowheads). To gain insight into the ability of AP maturation, double labeling of LC3 and LAMP2, an important lysosomal marker, was used to investigate fusion event between APs and lysosomes^47^. Compared with G93A-Con group, the percentage of colocalization of LC3 and LAMP2 was mainly reduced in MNs of G93A-Sim group (Fig. 4B, arrowheads). In agreement with these results, immunofluorescence staining in vitro also showed a significant reduction in the structure of LAMP2- and LC3- double positive in NSC34-hSOD1^G93A^ cells after simvastatin treatment (Fig. 8B and S1B), but no significant effect on the colocalization of P62 and LC3 (Fig. 8A and S1A). Together, these results implied that simvastatin could block the process of APs fusion with lysosomes in MNs of SOD1^G93A^ mice.

### Simvastatin repressed recruitment of Rab7 to membranes in MNs of SOD1^G93A^

**Fig. 5:**
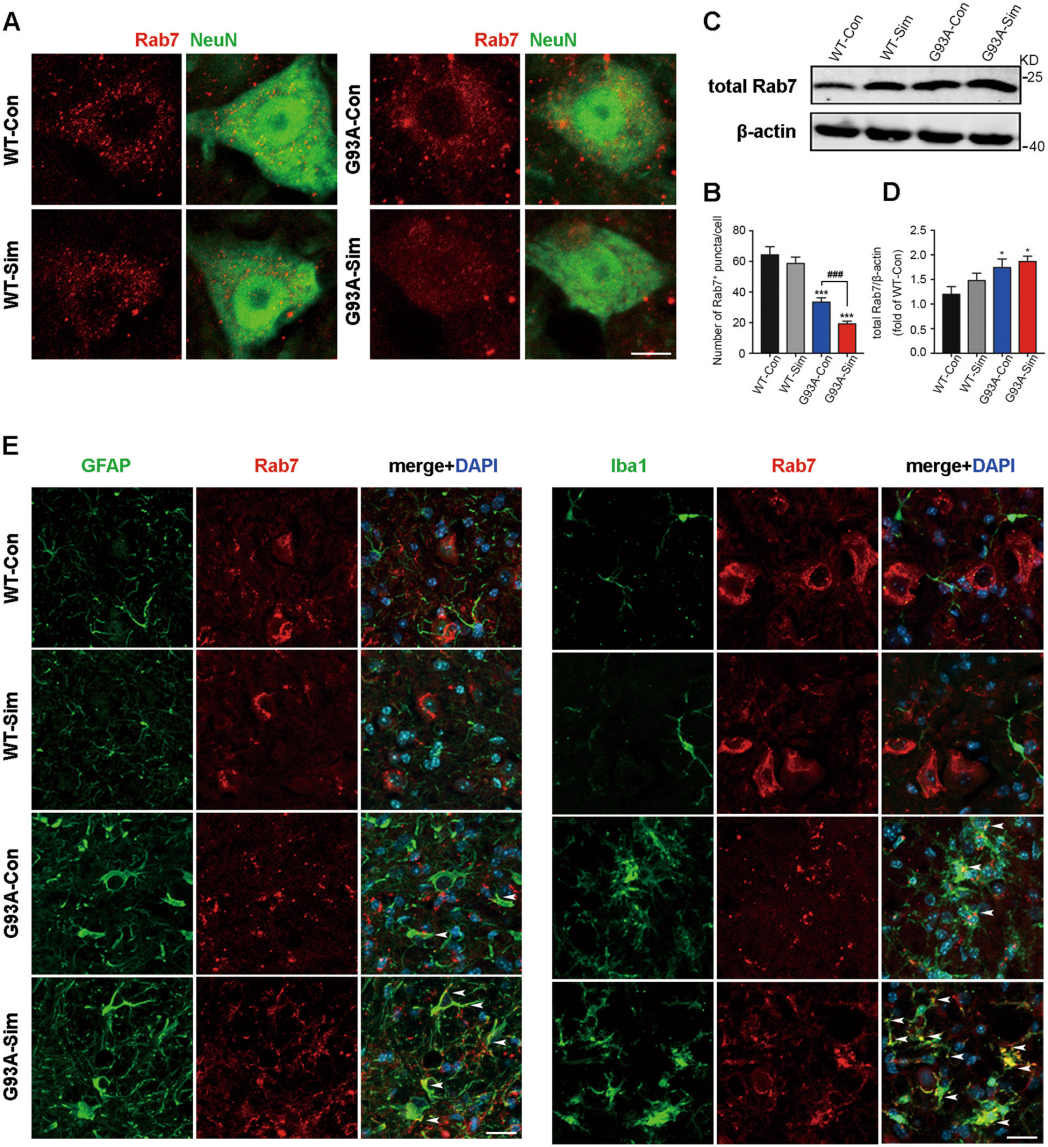
Simvastatin blocked Rab7 localization to membranes in MNs from SOD1^G93A^ mice. **A** Immunofluorescence labeling of Rab7 (red) in NeuN-positive motoneurons (green) at 120 days (Scale bars, 10 μm). **B** Quantitative analysis of Rab7-positive puncta. **C** Western blot analysis of total Rab7 in the lumbar spinal cord at 120 days. **D** Quantification of total Rab7 levels. **E** Immunostaining of Rab7 (red) and GFAP (green), Rab7 (red) and Iba1 (green) in the lumbar spinal cord at 120 days (Scale bars, 20 μm). Nuclei were stained with DAPI (blue). White arrowheads show the portions of GFAP^+^/Rab7^+^ and Iba1^+^/Rab7^+^, respectively. (Data represent the mean ± SEM, *n* = 5 mice per group; statistical significance was assessed by one-way ANOVA or an unpaired *t*-test, *P ≤ 0.05, **P ≤ 0.01, ***P ≤ 0.001, ^#^*P* ≤ 0.05, ^##^*P* ≤ 0.01, ^###^*P* ≤ 0.001).

Fusion events between APs and lysosomes play a key role in maintaining of autophagy flux balance. It is generally considered that Rab7 has functions in the maturation of late autophagic vacuoles^48,49^. Firstly, we investigated Rab7 localization to membrane via immunofluorescence, and the amount of Rab7-positive structures was ~50% less after simvastatin administration compared with SOD^G93A^ mice, but there was no significant difference between WT-Con and WT-Sim groups (Fig. 5A, B). Paradoxically, western blot analysis indicated that the level of total Rab7 protein in cell was increased in both G93A-Con and G93A-Sim mice. We also noted a slight upregulation of Rab7 in the latter, but no significant statistical difference in the indicated two groups (Fig. 5C, D). To explain these results, we double stained for Rab7 and GFAP or Iba1. Compared to WT, increased colocalization of Rab7 and GFAP or Iba1 was seen in SOD1^G93A^ mice, especially after simvastatin treatment (Fig. 5E). Meanwhile, Rab7 constructs were mainly visible in NeuN-positive cells of WT mice (Fig. 5A).

**Fig. 6:**
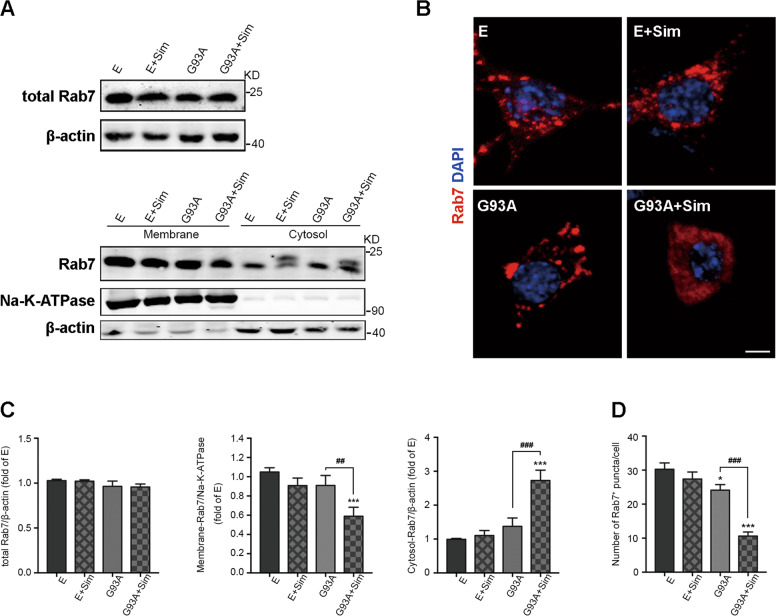
Simvastatin inhibited Rab7 localization to membranes in NSC34-hSOD1^G93A^ cells. **A** The expression of Rab7 protein in total cell, membranes, cytosol for each group by western blot analysis. NSC34-E and NSC34-hSOD1^G93A^ cells were incubated with Simvastatin (1 µM) for 24 h. **B** Rab7-positive structures (red) in NSC34-E and NSC34-hSOD1^G93A^ cells treated or untreated with simvastatin (1 µM) for 24 h. Nuclei were stained with DAPI (blue, Scale bars, 5 μm). **C** Quantification of Rab7 protein in total cell, membranes, cytosol for each group. **D** Quantitative analysis of intracellular Rab7 puncta in each group. (Data represent the mean ± SEM, *n* = 20 cells per group; statistical significance was assessed by one-way ANOVA or an unpaired *t*-test, *P ≤ 0.05, **P ≤ 0.01, ***P ≤ 0.001, ^#^*P* ≤ 0.05, ^##^*P* ≤ 0.01, ^###^*P* ≤ 0.001).

To understand phenomenon of Rab7-positive structures, we also validated localization of Rab7 to membranes in vitro. Quantitative analysis of western blot suggested that level of total Rab7 protein was not significantly different among the groups (Fig. 6A, top row, 6C). Active Rab7 peripherally binds to membranes via protein prenylation. We next detected the abundance of Rab7 in membrane and cytosolic fractions by western blot. The results revealed that the fraction of membrane-associated Rab7 was extensively reduced in the NSC34-hSOD1^G93A^ cells treated with simvastatin while the fraction of Rab7 in the cytoplasm was distinctly elevated, but unaltered in NSC34-E cells (Fig. 6A, below row, 6C). Moreover, compared with NSC34-hSOD1^G93A^, Rab7-localized structures were approximately invisible throughout the total body of simvastatin-treated NSC34-hSOD1^G93A^ cells, but there was no significant alteration in NSC34-E with or without simvastatin (Fig. 6B, D). Based on these results demonstrated that simvastatin repressed formation of membrane-associated Rab7 in MNs of SOD1^G93A^.

**Fig. 7:**
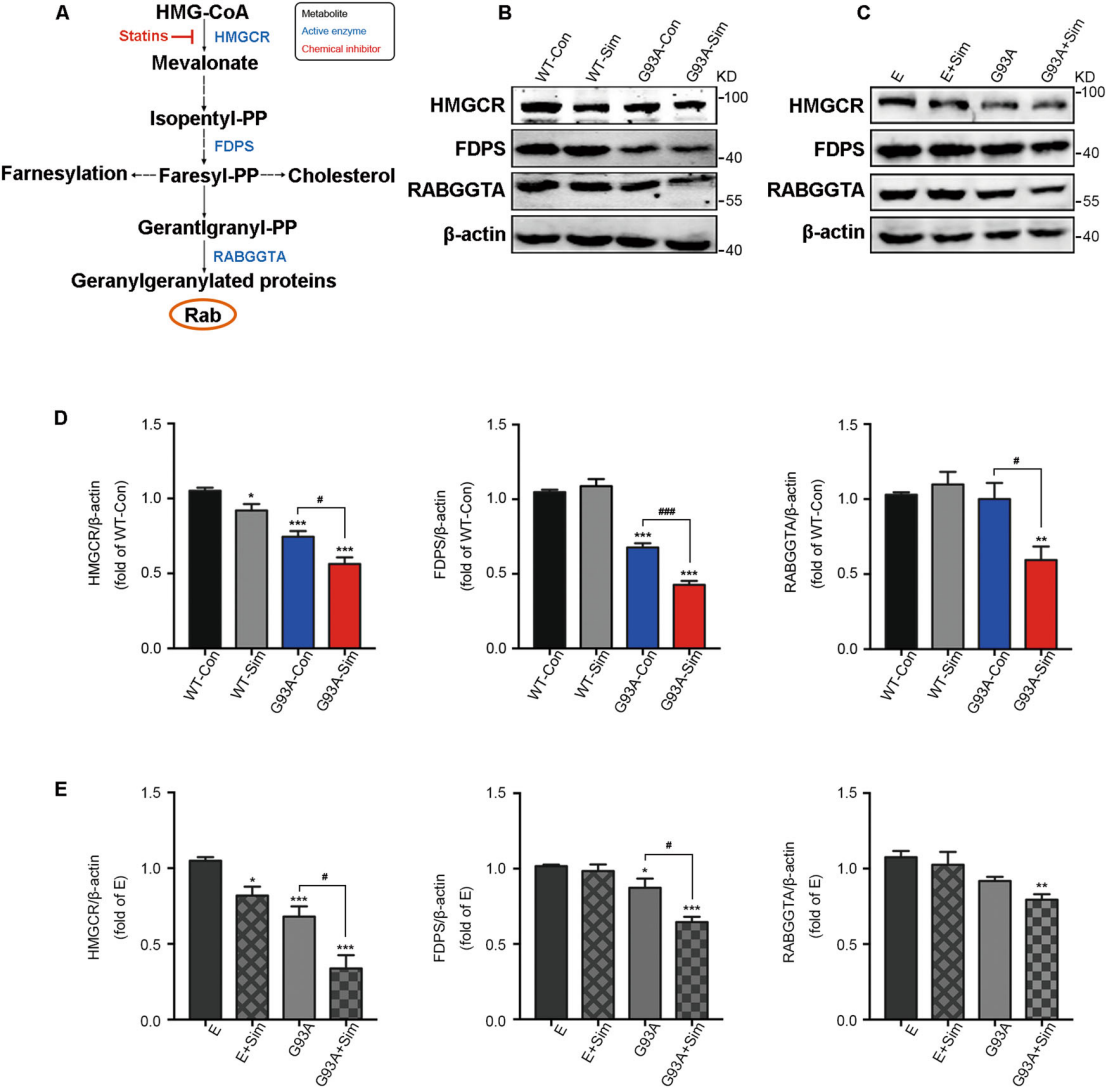
Effect of simvastatin on the mevalonate pathway in SOD1^G93A^ mice and NSC34-hSOD1^G93A^ cells. **A** Schemetic of the mevalonate pathway. **B** Western blot analysis of levels of HMGCR, FDPS, and RABGGTA in the lumbar spinal cord at 120 days. **C** Western blot analysis of the levels of HMGCR, FDPS, and RABGGTA in each group. NSC34-E and NSC34-hSOD1^G93A^ cells were treated with Simvastatin (1 µM) for 24 h. **D** Quantitative analysis of expression of HMGCR, FDPS, and RABGGTA in WT and SOD1^G93A^ mice treated or untreated with simvastatin respestively. **E** Quantitation of HMGCR, FDPS, and RABGGTA in NSC34-E and NSC34-hSOD1^G93A^ cells treated or untreated with simvastatin. (Data represent the mean ± SEM, *n* = 5 mice or 20 cells per group; statistical significance was assessed by one-way ANOVA or an unpaired *t*-test, *P ≤ 0.05, **P ≤ 0.01, ***P ≤ 0.001, ^#^*P* ≤ 0.05, ^##^*P* ≤ 0.01, ^###^*P* ≤ 0.001).

The effect of isoprenoids on small GTPase protein Rab7 trafficking to membranes have been studied extensively in the past^19,50,51^. Next, to investigate the role of statins on key enzymes closely involved in FPP and GGPP synthesis in MVA pathway (Fig. 7A), we performed western blot analysis. Our date represented that all of HMGCR, FDPS, and RABGGTA that respectively catalyzed production of MVA, FPP, and GGPP were obviously decreased in the lumbar spinal cords of G93A-Sim mice compared with G93A-Con at 120 days (Fig. 7B, D). Similar results were found in NSC34-hSOD1^G93A^ cells (Fig. 7C, E). However, simvastatin only moderately reduced the level of HMGCR in both WT mice and NSC34-E cells and unaffected the expressions of FDPS and RABGGTA (Fig. 7B–E). Taken together, these data displayed the inhibitory effect of statin-mediated reduction in isoprenoids on Rab7 localization to membrane in MNs of SOD1^G93A^.

### FPP or GGPP rescued defect of late autophagic flux and ameliorated aggregation of the SOD1 protein in NSC34-hSOD1^G93A^ cells treated with simvastatin

**Fig. 8:**
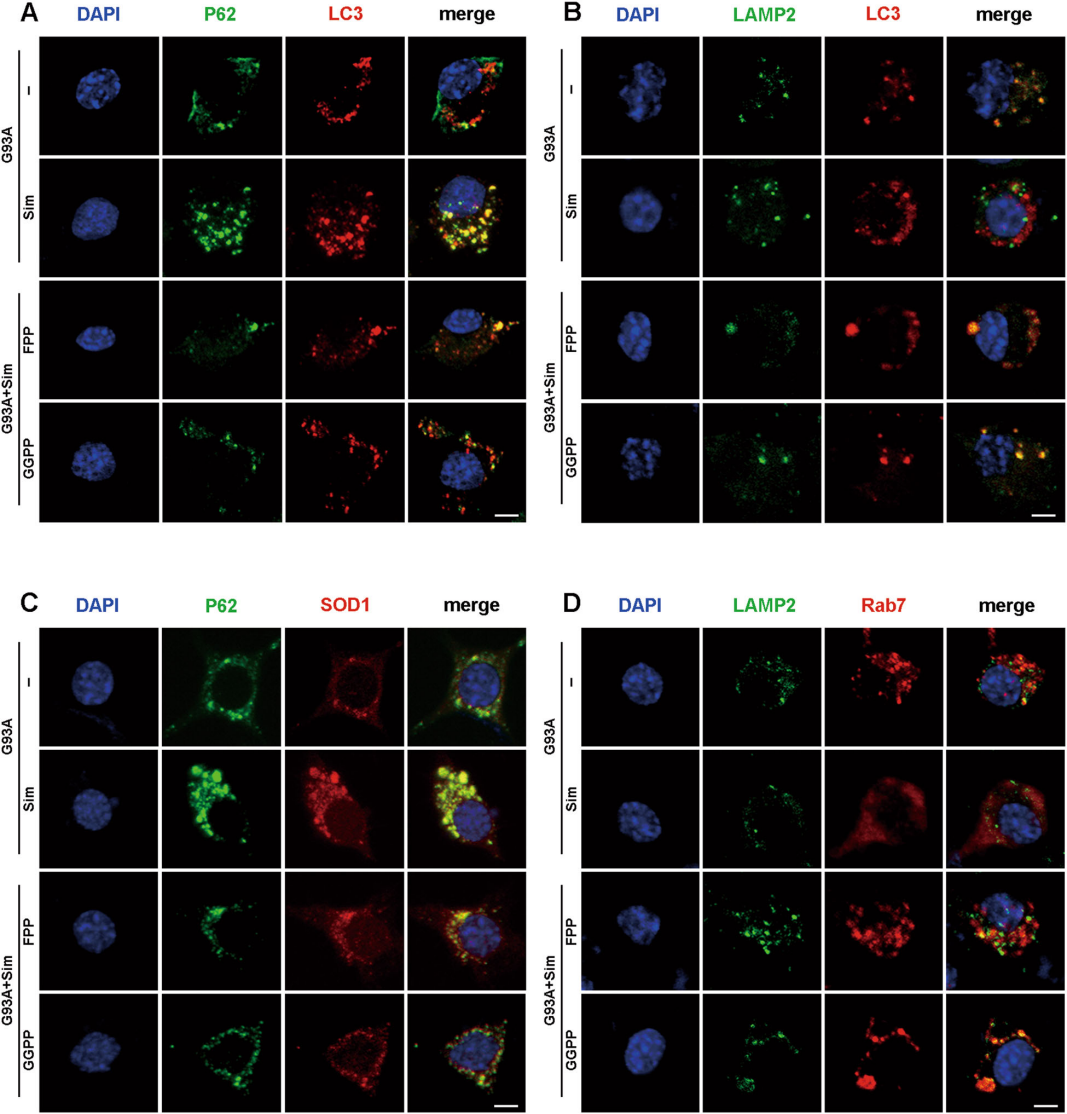
FPP or GGPP rescued defects of autophagic flux in simvastatin-treated NSC34-hSOD1^G93A^ cells. **A**–**D** Representative intensity projections from NSC34-hSOD1^G93A^ cells untreated or treated with the indicated chemicals: simvastatin (1 µM), simvastatin (1 µM) + FPP (10 µM) or simvastatin (1 µM) + GGPP (10 µM) for 24 h. Nuclei were stained with DAPI (blue). **A** Immunofluorescence labeling of P62 (green) and LC3 (red). P62- and LC3-double positive puncta indicating autophagosome formation (Scale bars, 5 μm). **B** Immunostaining of LAMP2 (green) and LC3 (red) in per cell group. Double positive structures for LAMP2 and LC3 representing fusion of autophagosomes with lysosomes (Scale bars, 5 μm). **C** Immunofluorescence labeling of P62 (green) and SOD1 (red) in per cell group (Scale bars, 5 μm). **D** Immunofluorescence labeling of LAMP2 (green) and Rab7 (red) in per cell group (Scale bars, 5 μm).

To further explore the possibility of autophagy balance maintained by sufficient isoprenoids, FPP or GGPP was added to simvastatin-treated NSC34-hSOD1^G93A^ cells. As shown in Fig. 8, FPP and GGPP decreased comparably accumulation of p62 and LC3 but their colocalisation (seen as yellow dots in the merge column) could not be affected (Figs. 8A and S1A). Consistent with Fig. 4, at the presence of simvastatin, colocalisation of LC3 and LAMP2 (yellow puncta) were drastically impeded leading to massive accumulation of LC3 and P62 in NSC34-hSOD1^G93A^ cells and finally aggreavated impairment of late autophagic flux (Figs. 8A, B and S1A, B). As we expect, colocalisation of LC3-positive APs to lysosomes marked by LAMP2-positive was significantly upregulated by simultaneous treatment of cells with simvastatin and FPP or GGPP. Thus, these events improved blockage of autophagic flux and eliminated aggregation of SOD1 in NSC34-hSOD1^G93A^ cells (Figs. 8A, B, C and S1A, B, D, E). Moreover, the inhibitory effect on activation of Rab7 was reversed by simvastatin combination with FPP or GGPP, which promoted relocation of active Rab7 to lysosomal membrane (Fig. 8D and S1C). Therefore, these results showed that inhibition of Rab7 activation caused by reduced isoprenoid, including FPP or GGPP, led to enhanced simvastatin-induced defect of late autophagic flux.

### Simvastatin worsened the disruption of APs fusion with lysosomes in NSC34-hSOD1^G93A^ cell via reduced prenylation of Rab7

**Fig. 9:**
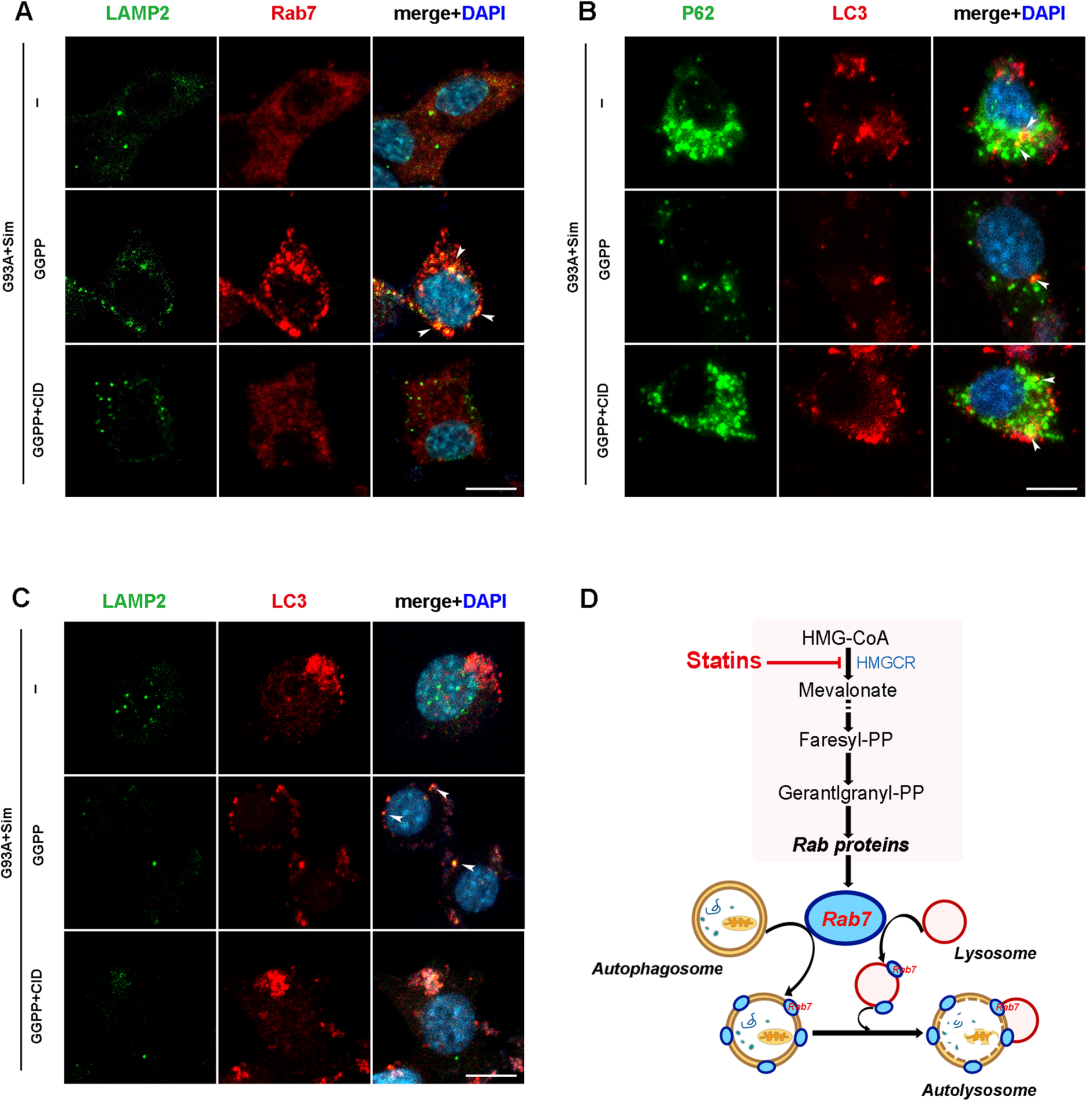
Inhibition of Rab7 recruitment to lysosome by CID1067700 aggravated defect of late autophagic flux in NSC34-hSOD1^G93A^ cells. **A**–**C** Simvastatin-treated NSC34-hSOD1^G93A^ cells were incubated with or without GGPP (10 µM) for 24 h, and were treated with GGPP (10 μM) for 24 h combined with CID1067700 (40 μM) for 2 h before cell harvesting. CID1067700, a late endosome GTPase Rab7 receptor antagonist. Nuclei were stained with DAPI (blue). **A** Immunofluorescence labeling of Rab7 (red) and LAMP2 (green) in per cell group. **B** Immunofluorescence labeling of LC3 (red) and P62 (green) in per cell group. **C** Immunofluorescence labeling of LC3 (red) and LAMP2 (green) in the per cell group. (Scale bars, 10 μm). **D** Schematic view of the potential mechanisms by which simvastatin inhibited Rab7-driven membrane fusion of autophagosomes with lysosomes, leading to aggravated impairment of late autophagic flux and accelerated neuron death.

To further understand the role of Rab7 on coordinating fusion between APs and lysosomes in NSC34-hSOD1^G93A^ cells after simvastatin incubation, we used CID1067700 (CID) to inhibit activity of Rab7. CID is a high-affinity and specific Rab7 inhibitor, which blocks the fusion of APs and lysosomes^52,53^. We thus investigated appearance of APs and Rab7 under CID administration both in NSC34-E (Fig. S2A) and NSC34-hSOD1^G93A^ cells (Fig. 9). Upon simvastatin treatment, colocalization of Rab7- and LAMP2- positive constructs was almost completely abolished. In line with Fig. 8, supplementation of GGPP had a great effect on shift of active Rab7 and LC3-positive APs to lysosomes (yellow puncta, Figs. 9A, C and S2B, C), after which accumulation of P62 and LC3 started to decrease (Fig. 9B). Importantly, we found that GGPP combination with CID treatment changed Rab7 behavior and blocked the fusion and degradation of LC3-positive APs with lysosomes, which was similar to the phenotype induced by simvastatin treatment alone (Figs. 9A, C and S2B, C). Consistently, inhibition of Rab7 led to re-accumulation of P62 and LC3 (Fig. 9B). However, under either condition colocalization of LC3 and P62 was not altered in line with Figs. 4 and 8. In addition, we detected level of cholesterol by Filipin staining. The results showed that simvastatin had no effect on the intracellular free cholesterol in both NSC34-E and NSC34-hSOD1^G93A^ cells even though NSC34-hSOD1^G93A^ cells have lower free cholesterol than NSC34-E cells, which could be determined by the phenotype of the NSC34-hSOD1^G93A^ cells (Fig. S1F). These experiments strengthened the notion that simvastatin functioned to suppress fusion of APs and lysosomes in NSC34-hSOD1^G93A^ cells mainly by interfering with Rab7 prenylation.

## Discussion

The MVA pathway, fundamental for cholesterol synthesis as well as production of non-sterols termed isoprenoids such as FPP and GGPP, is necessary for various metabolic pathways^19,54^. MVA pathway blockage has been associated to autophagy deficiency, possibly leading to cell death. The inhibitory function of statins on HMG-CoA reductase resulted in depletion of cholesterol and isoprenoids in MVA pathway^19,55^. Statins thus have therapeutic application in diseases like inflammatory disease, cancer, and neurodegenerative disease including AD, PD, and MS, probably due to its pleiotropic effects independent of lowering cholesterol levels^24–26,56^. However, impacts of statins on ALS have gained less attention. In present study, upon simvastatin treatment we evaluated autophagic performance in SOD1^G93A^ mice that had been considered as a classic ALS model^57^. Expectedly, our date showed that simvastatin worsened defective autophagic flux causing increased accumulation of APs in SOD1^G93A^ mice (Fig. 1). Additionally, we found that massive loss of MNs resulted in deterioration of disease progression, which could be highly linked to cytotoxicity effect of abnormal SOD1 aggregation mediated by deficient autophagy (Figs. 2 and 3). An important pathological feature of patients with ALS is that a large number of malformed proteins gathered in MNs leading to neuronal degeneration and death^40,42,43^. Thus, it is possible that simvastatin used in clinical practice in ALS may play a negative role.

Autophagy can be envisaged as a process to consist of autophagosomal formation, targeted transport, and fusion with lysosome that degraded cytosolic components engulfed in autophagic membranes^7,8^. AP formation is facilitated by p62 interacting LC3 to encase pathogens in APs^3^. Our results demonstrated that the ability of p62 binding to LC3 was not changed by simvastatin treatment (Fig. 4). We speculated that simvastatin had no impact on APs formation in the lumbar spinal cords MNs of SOD1^G93A^ mice. Interestingly, reduced colocalization of LC3 and LAMP2 could reflect impaired fusion of APs with lysosomes, which may explain why simvastatin worsened defects of autophagic flux (Fig. 4).

It has been shown recently that prenylation of Rab small GTPases appears to be one of important mechanisms involved in autophagy regulation^19^. Among these, location of prenylated Rab7 to membrane is responsible for AP maturation and facilitating AP fusion to lysosome^9–11^. Here, we noted that simvastatin severely decreased Rab7 localization to the membrane in MNs of SOD1^G93A^ both in vivo and in vitro. In contrast to, the activity of Rab7-binding membrane in WT mice and NSC34-E cells unaffected (Figs. 5 and 6). This may suggest that MNs of SOD1^G93A^ are more susceptible to be damaged by simvastatin. Additionally, we also found that Rab7 were mainly located in Iba1- and GFAP-positive cells in SOD1^G93A^ mice, especially after simvastatin treatment. This may provide a sound explanation for upregulation of total Rab7 protein in the lumbar spinal cords of SOD1^G93A^ mice with or without simvastatin whereas in vitro experiments do not (Figs. 5 and 6). However, the role of Rab7 in astrogliosis and microgliosis in spinal cord of SOD1^G93A^ mice remains unknown.

Isoprenoids have been shown to be essential for protein prenylation, which are required for proper function of membrane-localized Rab and for normal autophagy^19,58^. In our previous work it has been shown that simvastatin aggravated autophagic disruption through reduction of isoprenoids in NSC34-hSOD1^G93A^ cells^30^. In present study, we further saw that simvastatin extensively decreased the fraction of Rab7 associated with the membrane while increased the fraction of Rab7 in the cytoplasm, but unaltered in NSC34-E cells (Fig. 6). Statin has been shown to inhibit MVA pathway. We found that expression levels of key enzymes linked to isoprenoids production in MVA pathway had been diminished after Simvastatin treatment (Fig. 7). Decreased prenylation of Rab7 have been reported to impair autophagy^50,51^. Our current study demonstrated that anchor of Rab7 to lysosome has been effectively rescued with supplementation of FPP or GGPP in Simvastatin-treated NSC34-hSOD1^G93A^ cells (Figs. 8 and S1). Consistent with the fact that lack of FPP or GGPP associated with reduced autophagic flux^30^, supplement of FPP or GGPP reversed largely inhibitory effect of statin on autophagasomes fusion with lysosomes and further promoted degradation of SOD1 (Figs. 8 and S1). It is known that activity of Rab7 binding to lysosomal membrane is specifically blocked by CID1067700^52,53^. We also observed that addition of GGPP was still unable to rescue late stage of autophagic flux defects induced by simvastatin under the condition of CID1067700 (Figs. 9 and S2). Overall, we proposed one possible mechanism in NSC34-hSOD1^G93A^ cells wherein statins, inhibiting synthesis of isoprenoids and decreasing protein prenylation, could block Rab7 location to lysosome and repress autophagic flux. Notably, a widely accepted view is the pleiotropic effects of statin associated with lack of prenylation but independent of reduction of cholesterol^59,60^. Recent study reported that free cholesterol was unaltered in atorvastatin-treated neurons^61,62^. Similar to previous studies, our results showed that simvastatin unaltered the level of free cholesterol in NSC34-hSOD1^G93A^ cells (Fig. S1F).

Our results exposed that simvastatin inhibited isoprenoids synthesis in the MVA pathway, which further impeded prenylation of Rab7 accompanied by inhibitory localization of Rab7 to lysosomal membrane as well as deteriorated blockage of autophagic flux at late stage. In this case, simvastatin ultimately resulted in massive neuronal death and earlier onset of the disease phenotype in SOD1^G93A^ mice. The present study together with our previous findings suggested the potential risk of statin-induced neuronal death. Therefore, a caution needs to be payed for further clinic application of statins in ALS.

## Supplementary information

Figure S1

Figure S2

## Data Availability

The data generated and analyzed in this study are available from the corresponding authors upon request.
