# Correction: Simvastatin accelerated motoneurons death in SOD1^G93A^ mice through inhibiting Rab7-mediated maturation of late autophagic vacuoles

**DOI:** 10.1038/s41419-025-07349-x

**Published:** 2025-02-27

**Authors:** Lin Bai, Yafei Wang, Jia Huo, Shuai Li, Ya Wen, Qi Liu, Jing Yang, Yaling Liu, Rui Li

**Affiliations:** 1 Department of Neurology, The Second Hospital of Hebei Medical University, Shijiazhuang, Hebei, 050000 P.R. China; 2 Neurological Laboratory of Hebei Province, Shijiazhuang, Hebei, 050000 P.R. China

Correction to: *Cell Death & Disease* 10.1038/s41419-021-03669-w, published online 12 April 2021

In the published article, one error appeared in the Fig. 1A owing to our negligence during editing of image. The other error was in Fig. 2B, both the WT-Con group and the WT-Sim group showed no aggregation of SOD1 by fluorescent staining so that the image of WT-con was very similar to that of WT-Sim. Due to our negligence, the same image was used for the WT-Con group and the WT-Sim group. We have revised the two errors and shown the correction images in the corrected file. All of our authors confirmed that this revision will not change our results and conclusions. The authors would like to assure readers that the corrected values and labels do not alter the interpretations or validity of the research.

**Error Correction**

1. Figure 1A: We checked the original images again and found that it was indeed an error for the *β-actin* image in Fig. 1A owing to our mistake during the figure editing process (As shown in the red box).
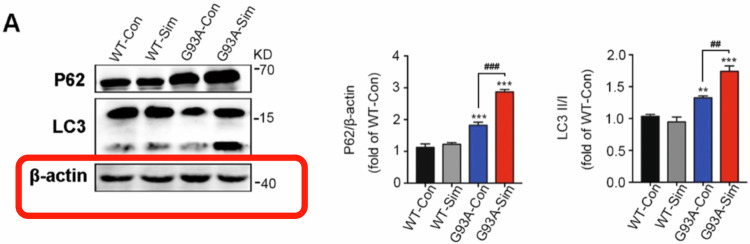


**Published Figure1A**

Original image of Western Blot for Fig. 1A.

Western blot analysis of P62 in the lumbar spinal cords of WT and SOD1G93A mice treated with or without simvastatin at 120 days.
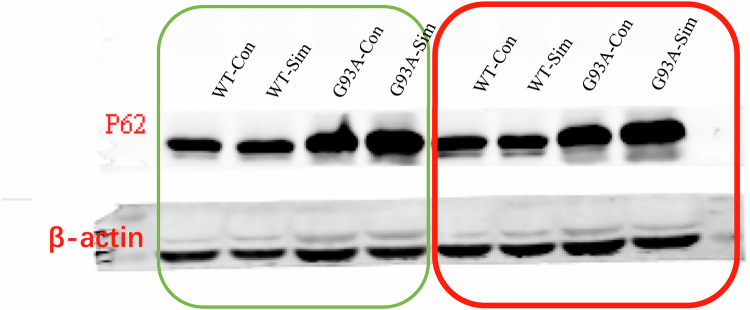


Eight bands represent two independent experiments (marked with red and green boxes respectively) The order of the samples is WT-Con, WT-Sim, G93A-Con, G93A-Sim. The red box represents the *β-actin* image that correctly corresponds to the P62 image shown in Fig. 1A.

**Corrected Figure 1A** 
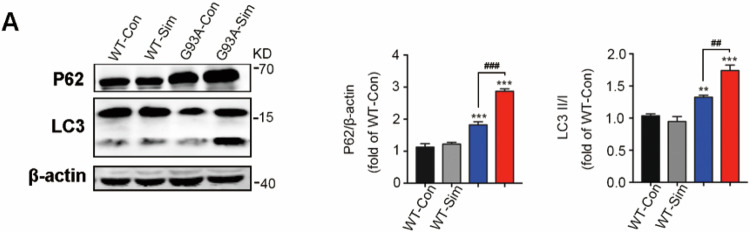


**Corrected Figure 1** 
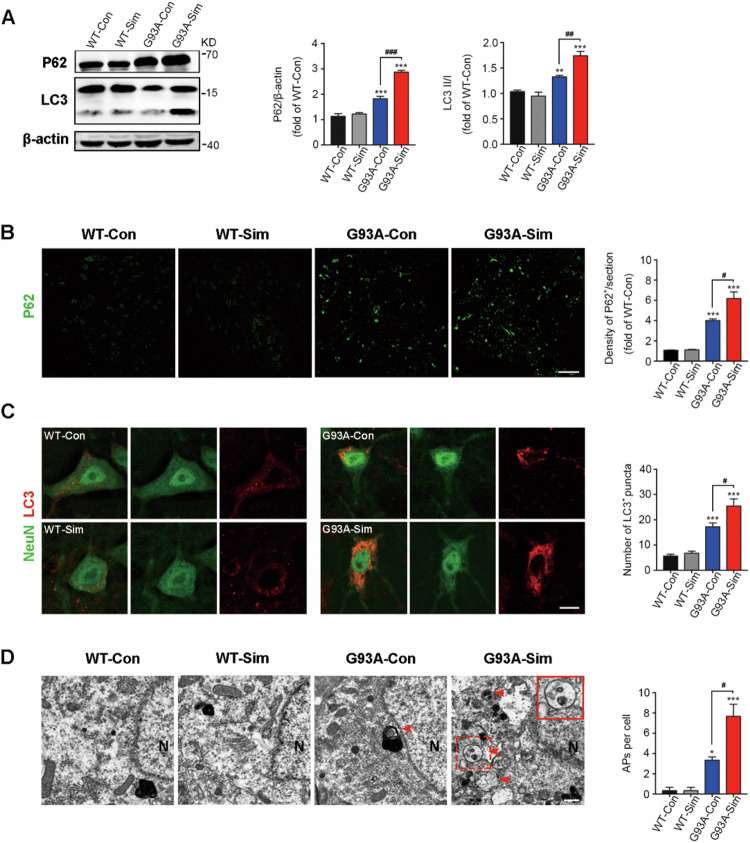


2. Figure 2B: Due to our negligence, the same image was used for the WT-Con group and the WT-Sim group (As shown in the red box).
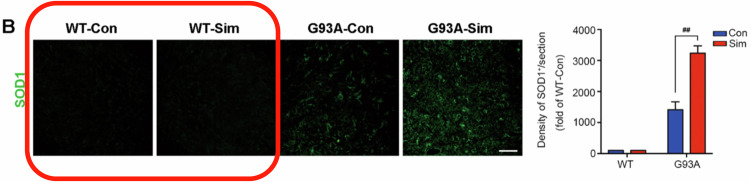


**Published Figure2B**

Original image of Immunofluorescence labeling for Fig. 2B.

Immunofluorescence labeling of SOD1 (green) in the lumbar spinal cord at 120 days.

WT-Con
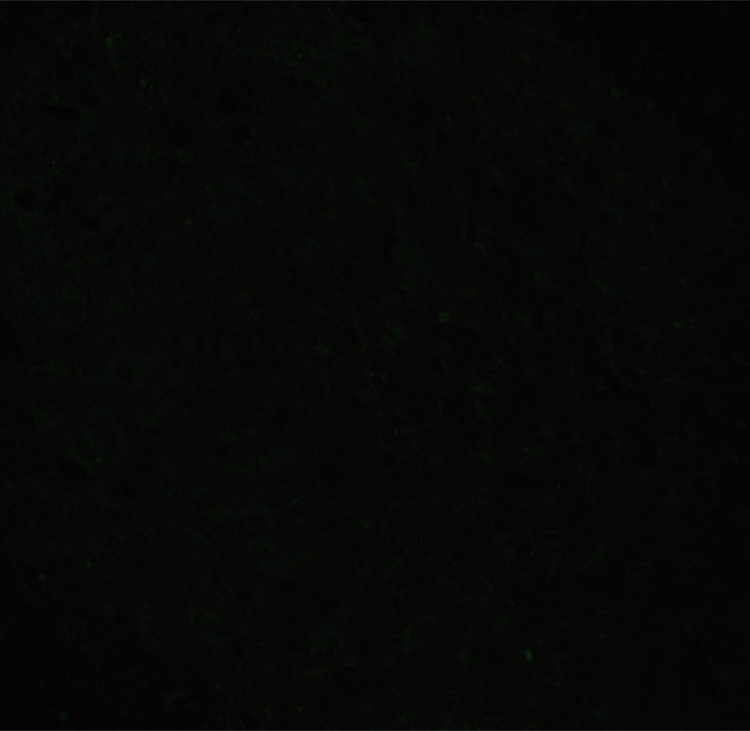


WT-Sim
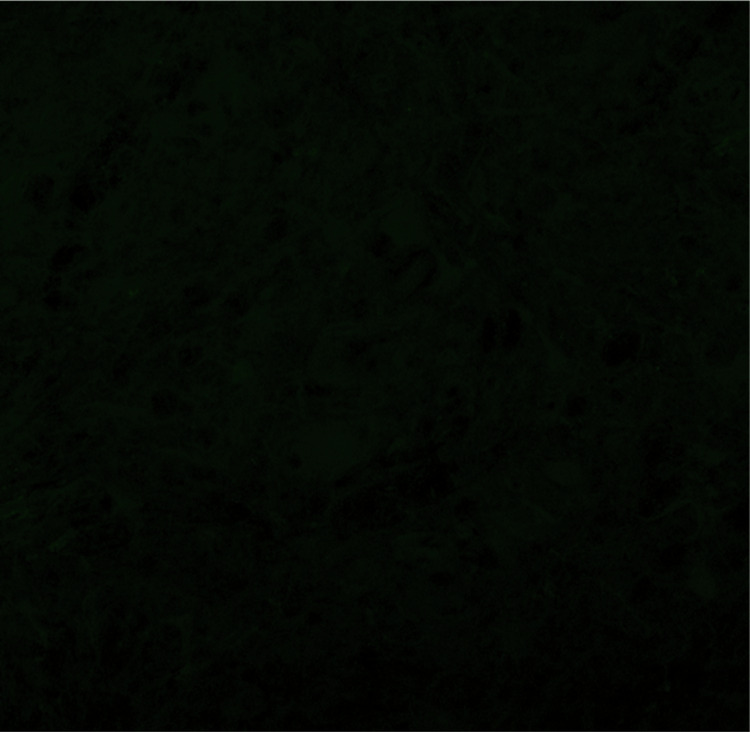


In WT group (WT-Con and WT-Sim), there was no accumulation of SOD1 by fluorescent staining, and the image showed mainly background.

**Corrected Figure 2B** 
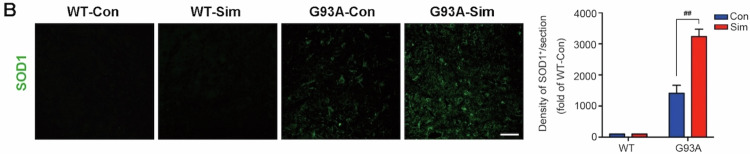


**Corrected Figure 2** 
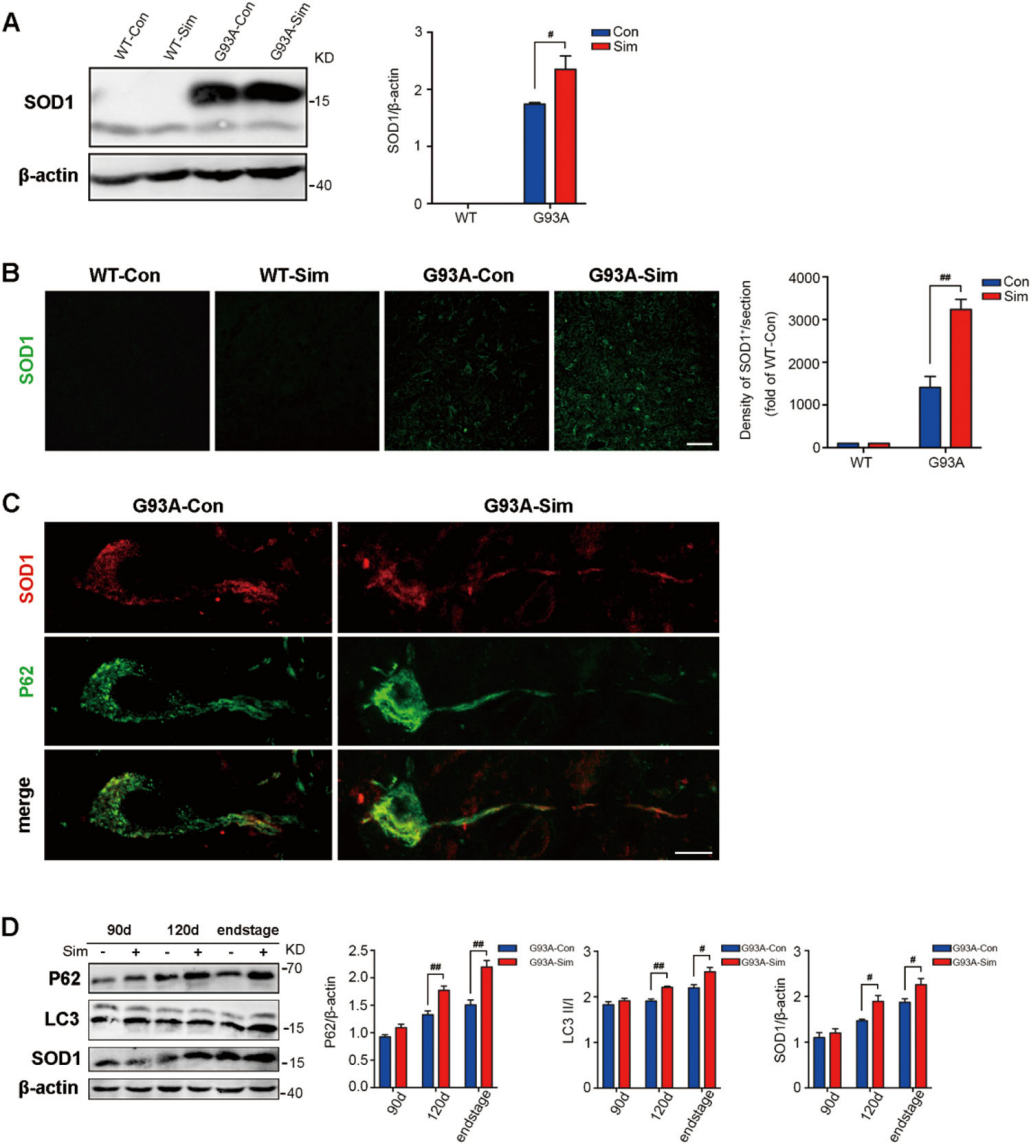


The original article has been corrected.

