# Supplementary figures and images for: Simvastatin accelerated motoneurons death in SOD1G93A mice through inhibiting Rab7-mediated maturation of late autophagic vacuoles
Source: Cell Death Dis. 2021 Apr 12;12(4):392. doi: 10.1038/s41419-021-03669-w (PMC8041862; doi:10.1038/s41419-021-03669-w)

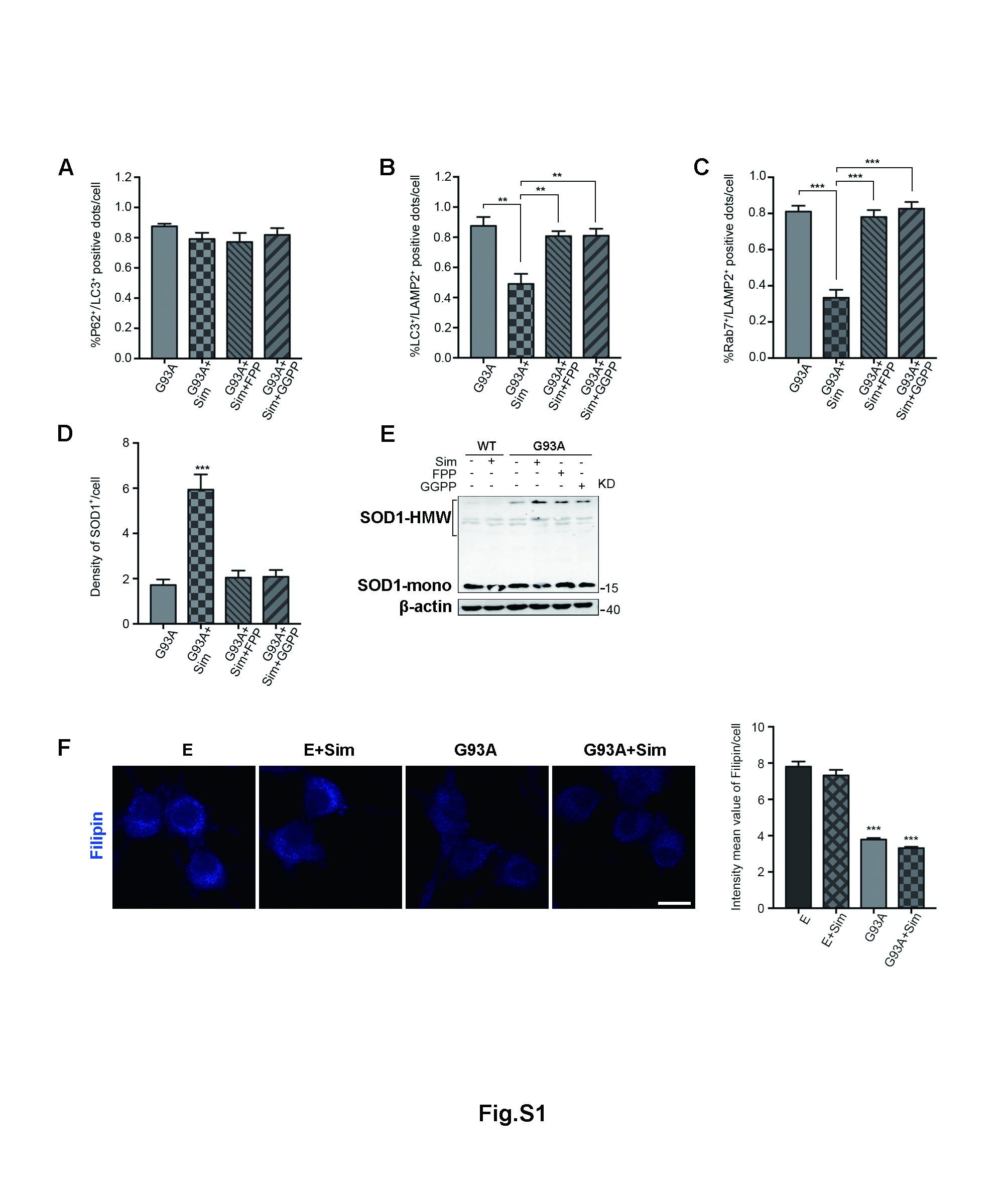

Supplement: Supplementary file 1 — Figure S1 [file 41419_2021_3669_MOESM1_ESM.tif]

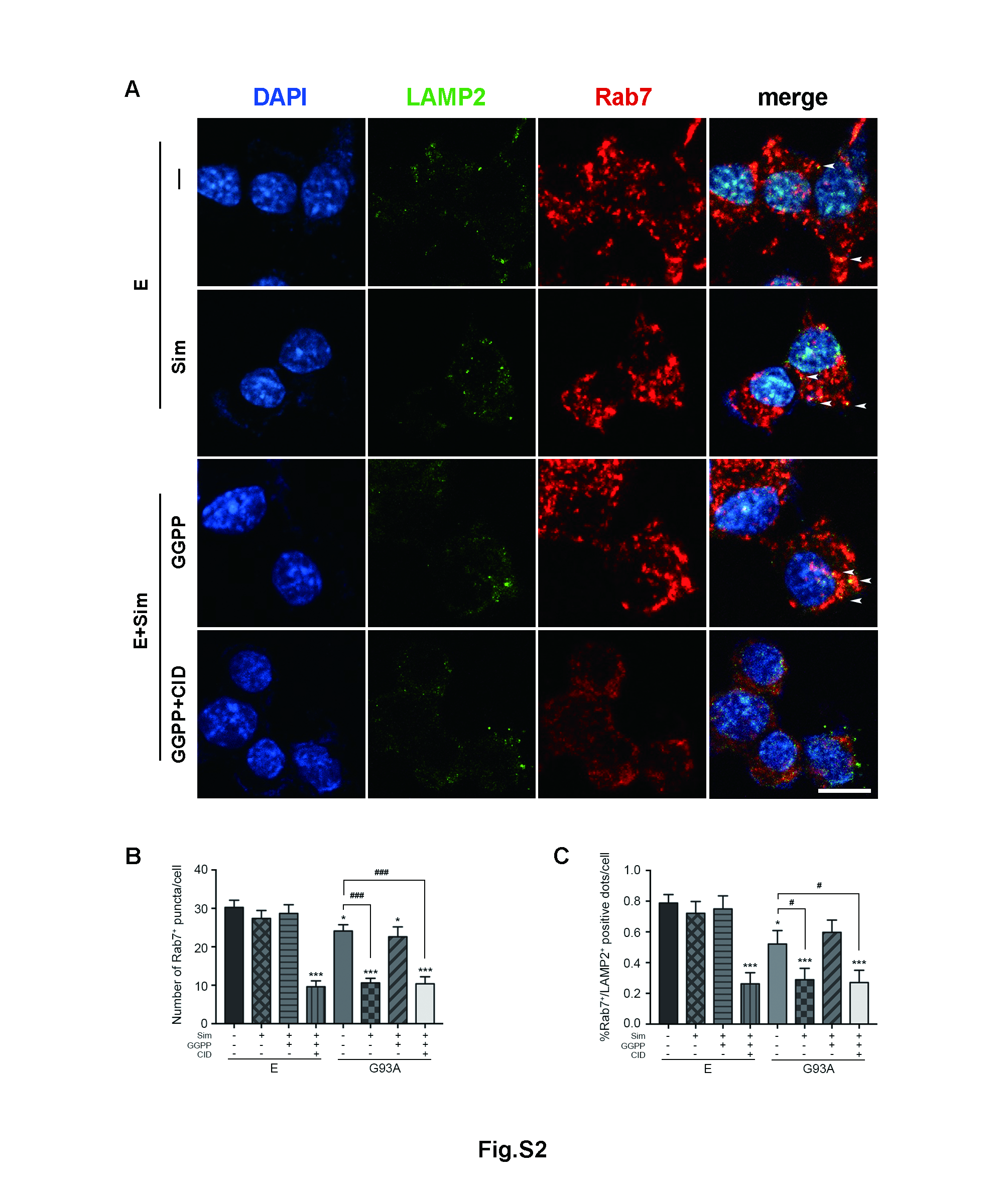

Supplement: Supplementary file 2 — Figure S2 [file 41419_2021_3669_MOESM2_ESM.tif]
